# Trends in cardiorespiratory fitness among apparently healthy adults from the Ball State Adult Fitness Longitudinal Lifestyle STudy (BALL ST) cohort from 1970–2019

**DOI:** 10.1371/journal.pone.0242995

**Published:** 2020-12-01

**Authors:** Matthew P. Harber, McKenzie Metz, James E. Peterman, Mitchell H. Whaley, Bradley S. Fleenor, Leonard A. Kaminsky

**Affiliations:** 1 Clinical Exercise Physiology, Ball State University, Muncie, Indiana, United States of America; 2 Fisher Institute of Health and Well-Being, Ball State University, Muncie, Indiana, United States of America; University of Calgary, CANADA

## Abstract

**Introduction:**

Cardiorespiratory fitness (CRF) is a strong independent predictor of cardiovascular disease (CVD) and CVD mortality. However, little is known in regards to how CRF has trended in apparently healthy adults over the past several decades.

**Purpose:**

To analyze trends in CRF and CVD risk factors over the last 50 years in a population of apparently healthy adult men and women.

**Methods:**

Participants were 4,214 apparently healthy adults (2,390 men and 1,824 women) from the Ball State Adult Fitness Longitudinal Lifestyle STudy (BALL ST) that performed maximal cardiopulmonary exercise testing between 1970–2019 for the assessment of CRF defined as VO_2max_ (ml/kg/min). Participants were self-referred either to a community-based exercise program, fitness testing, or were research subjects in exercise related studies and were placed into groups by decade based on testing date.

**Results:**

CRF showed a general trend to decline (P<0.05) from the 1970s to the 2000s with an increase (P<0.05) from the 2000s to the 2010s for both men and women. This pattern persisted for age and sex-adjusted CRF level, determined by Fitness Registry and the Importance of Exercise: A National Data Base (FRIEND). For both women and men, CRF across the decades was associated (P<0.05) with the prevalence of physical inactivity, smoking, obesity, dyslipidemia and hypertension, and with diabetes in men only.

**Conclusion:**

CRF declined from 1970 through the 2000s in a cohort of apparently healthy men and women which was associated with worsening CVD risk profiles. However, the decline in CRF was attenuated over the past decade which may have a positive impact on future CVD in the population. Promoting physical activity to increase CRF should be a primary aspect of CVD prevention programs.

## Introduction

Cardiorespiratory fitness (CRF) is a measure of functional capacity and aerobic power (i.e., VO_2max_) that involves the integrative relationship of multiple physiological systems to consume and transport oxygen to metabolically active tissues performing work. While CRF was initially investigated for its relation with human performance, over the past 30 years CRF has been established as a powerful determinant of cardiovascular disease (CVD) and CVD-mortality that is independent of traditional risk factors [1–4]. Based on the abundant evidence supporting the importance of CRF as a risk factor for CVD, the American Heart Association recently advocated that CRF be considered a clinical vital sign that is routinely measured in patient care [5].

Several investigations have reported temporal trends in CRF over the last several decades with conflicting results, which may be attributed to diverse cohort characteristics and various methods of assessing CRF. Two recent systematic reviews collectively including over 3 million individuals have reported a worldwide decline in CRF in both children [6] and adults [7] since 1980, which the authors suggest has contributed to a decline in population health. These findings have been supported by trends of declining CRF in New Zealand children [8], Canadian adults [9], and in patients referred for exercise testing at Duke University in the United States [10]. Contrary to these reports are analyses from the Cooper Clinic showing a general upward trend in CRF from 1970 to the early 2000s for both men and women [11, 12]. In addition to varying population characteristics, it should be noted these studies estimated CRF from field tests [6–8], submaximal [7, 9], or maximal exercise tests [10–12], which have substantial prediction error compared to the gold-standard assessment of CRF, maximal cardiopulmonary exercise testing (CPET) [13].

The Ball State Adult Fitness Longitudinal Lifestyle STudy (BALL ST) cohort is the largest known single-site database of CPET-derived CRF in apparently healthy adult men and women dating back over 50 years, which affords a unique opportunity to examine CRF trends over time. Importantly, we have previously established that CPET-derived CRF is predictive of CVD mortality in this cohort and that CPET-derived CRF is a more powerful predictor of CVD mortality than estimated measures of CRF [3]. The primary objective of the current study was to examine cross-sectional trends in CPET-derived CRF in apparently healthy adult men and women over the past five decades. A secondary purpose was to examine trends in CVD risk factors over the same timeframe. To achieve these aims, we examined CRF values and CVD risk factors from the BALL ST cohort between 1970–2019, hypothesizing that CRF would decline over time and that this decline would be associated with a worsening CVD risk factor profile.

## Materials and methods

This investigation was a cross-sectional analysis of 4,214 apparently healthy adults (1824 women, 2390 men) from the BALL ST cohort assessed between January 1, 1970 and December 31, 2019. Participants were self-referred to the Ball State University-Adult Physical Fitness Program, a community-based exercise program, for exercise testing, or were research subjects in clinical exercise physiology related studies who gave written informed consent for their data to be used for research. Participants were considered apparently healthy, as all were free from known CVD (history of cardiac arrest, coronary artery disease, heart failure, myocardial infarction, and stroke), chronic obstructive pulmonary disease, and cancer. This study was reviewed by the Ball State University Institutional Review Board and determined exempt as only de-identified data were used.

### Assessment of CRF

All participants performed a maximal CPET on a treadmill to assess VO_2max_ as a direct measure of CRF using a standardized or individualized protocol designed to achieve maximal effort within 8 to 12 minutes based on their physical activity level and other demographics [14]. Only the initial CPET was used for data analysis for individuals that had more than one test. A thorough description of the procedures used to assess CRF have been described previously [3, 14]. Measurements of gas exchange were performed throughout the CPET. As described in [14], prior to 1981, gas exchange analysis was conducted using a semiautomated system [15]. Between 1981–1991, gas exchange analysis was performed with an Applied Electrochemistry S3A O2 analyzer (Sunnyvale, CA), a Beckman CO2 analyzer (Fullerton, CA), and a Parkinson-Cowan gas meter (Manchester, England) which were integrated via an A-D converter (Rayfield Equipment; Waitsfield, VT) to an Apple IIe microcomputer. From 1992–2002 a Sensor Medics 2900 system (Yorba Linda, CA) was used and a Parvo metabolic testing system (Parvo Medics, Salt Lake City, Utah) was used from 2002–2019. Standardized procedures were followed for metabolic cart calibration and all tests were supervised by trained clinical exercise physiologists, with additional medical supervision when appropriate. Participants exercised to volitional fatigue and a respiratory exchange ratio (RER) of ≥1.0 was used as an objective indicator of maximal effort after completion of the test. VO_2max_ was determined by averaging the highest 2 to 3 consecutive VO_2_ values during a 30 second sampling period within 2 ml/kg/min occurring in the last 2 minutes of the test. Sex- and age-specific CRF percentiles were generated based on the Fitness Registry Importance of Exercise National Database (FRIEND) [16]. Age-predicted maximal heart rate was determined as 220 –age in years [17] and was >99% on average for each decade.

### Clinical measurements

All participants were instructed to refrain from exercise, caffeine, and alcohol for at least 12 hours before testing and arrive in a fasting state. Participants completed a health history questionnaire that included demographic information, personal and family medical history, medication usage, and lifestyle behaviors (i.e., smoking and physical activity). Each participant then completed a series of assessments including anthropometric measurements (height and weight), resting heart rate and blood pressure, blood chemistry and resting 12-lead electrocardiography using standardized laboratory techniques to ensure consistency across the five decades as described previously [3, 14, 18]. Body weight was measured to the nearest 0.1kg using a mechanical scale (Health O Meter 400KL Physician Scale) or a digital scale (Health O Meter 349KLX) verified with calibrated weights according to manufacturer guidelines. Resting heart rate was determined via a manual 30 second pulse rate following 10 minutes of seated rest. Resting blood pressure values were obtained in the seated position following at least 5 minutes of rest. A minimum of two blood pressure measurements were recorded with additional measures taken if the initial two differed by more than 6/4 mmHg for systolic and diastolic pressures, respectively. These clinical measurements in combination with information from the health history questionnaire were used to determine the presence of CVD risk factors including: obesity, hypertension, dyslipidemia, physical inactivity, smoking, and diabetes, defined according to current accepted risk factor criteria [19]. A de-identified dataset was retrieved from the BALL ST cohort with the following variables: age, sex, test year, body weight, body mass index, systolic blood pressure, fasting total cholesterol, fasting blood glucose, smoking status, self-reported physical activity status, CRF, respiratory exchange ratio, and health history questionnaire.

### Statistical analysis

SPSS V. 24 was used for all statistical analyses. Participants were placed into one of five groups based on year of testing (1970s = 1970–1979; 1980s = 1980–1989; 1990s = 1990–1999; 2000s = 2000–2009; 2010s = 2010–2019). Univariate analysis of variance (ANOVA) was used to test for differences between groups for each demographic characteristic (age, BMI, blood glucose, total cholesterol, systolic blood pressure) and exercise test variables (absolute VO_2max_ [L/min], relative VO_2max_ [ml/kg/min], FRIEND percentile, maximal heart rate). Men and women were examined independently. In the presence of a main effect, group differences were identified with pairwise comparisons using Bonferroni adjustment for multiple comparisons. Additionally, an analysis of covariance was performed to control for age differences in the groups when comparing changes in CRF across decades. Trends for CRF across decade were assessed using a general linear model and trends for prevalence of risk factors and medication usage were assessed with the Cochran-Armitage test. Multiple regression was used to examine the relationship between CRF and the CVD risk factors across the decades. Significance level was set at P < 0.05 and data are presented as mean ± standard deviation.

## Results

### Descriptive characteristics and CVD risk factors

Age, BMI, fasting blood glucose, fasting blood total cholesterol, and resting systolic blood pressure women and men are presented in Table 1. The prevalence of obesity, hypertension, diabetes, dyslipidemia, smoking, physical inactivity, and medication usage is presented in Table 2. For women, age was lower (P<0.05) in 1970s compared to the other decades and higher (P<0.05) in 2000s compared to the 1980s. BMI was lowest (P<0.05) in 1970s and increased (P<0.05) by decade through the 1990s. Prevalence of obesity increased (P<0.05) across decades from 1970s to 2000s (Table 2). Blood glucose was lower (P<0.05) in the 1970s compared to all other decades. Prevalence of diabetes was highest (P<0.05) in the 2000s and 2010s (Table 2). Total cholesterol was lower (P<0.05) in the 2010s compared to all other decades and was higher (P<0.05) during the 1970s and 1980s compared to the 1990s. Resting systolic blood pressure was lower (P<0.05) during the 2010s relative to the 2000s and 2010s. Prevalence of hypertension increased (P<0.05) from the 1970s to 1990s (Table 2).

**Table 1:** Descriptive characteristics and CVD risk factors for women (N = 1824) and men (N = 2390) by decade.

	1970s		1980s		1990s		2000s		2010s	
	n	Mean ± SD	n	Mean ± SD	n	Mean ± SD	n	Mean ± SD	n	Mean ± SD
**Women**										
Age (yr)	203	37.8 ± 10.8^b^^c^^d^^e^	532	41.6 ± 11.2^a^^d^	505	43.0 ± 12.4^a^	251	45.6 ± 13.2^a^^b^	333	43.5 ± 16.7^a^
BMI (kg/m^2^)	203	23.4 ± 4.3^b^^c^^d^^e^	532	25.5 ± 5.5^a^^c^^d^^e^	505	27.2 ± 6.2^a^^b^	251	28.2 ± 6.4^a^^b^	333	28.2 ± 7.2^a^^b^
Glucose (mg/dl)	133	83 ± 16^b^^c^^d^^e^	513	96 ± 21^a^	480	93 ± 16^a^	238	94 ± 19^a^	318	95 ± 21^a^
Total Cholesterol (mg/dl)	200	208 ± 39^c^^e^	517	205 ± 44^c^^e^	485	197 ± 38^a^^b^^e^	505	202 ± 37^e^	319	185 ± 39^a^^b^^c^^d^
SBP (mmHg)	203	118 ± 13	531	117 ± 14	505	119 ± 17^e^	249	119 ± 15^e^	330	114 ± 13^c^^d^
**Men**										
Age (yr)	544	40.5 ± 9.8^c^^d^	779	41.6 ± 10.6^c^^d^	514	43.9 ± 11.8^a^^b^^d^^e^	209	48.9 ± 13.2^a^^b^^c^^e^	344	41.1 ± 16.1^c^^d^
BMI (kg/m^2^)	544	25.9 ± 3.4^c^^d^^e^	779	26.3 ± 4.^c^^d^^e^	514	28.7 ± 5.2^a^^b^	209	29.5 ± 6.2^a^^b^^e^	344	28.1 ± 5.4^a^^b^^d^
Glucose (mg/dl)	279	89 ± 19^b^^c^^d^^e^	722	99 ± 19^a^	505	100 ± 24^a^	196	102 ± 31^a^	290	97 ± 25^a^
Total Cholesterol (mg/dl)	524	228 ± 46^b^^c^^d^^e^	733	217 ± 43^a^^c^^d^^e^	775	204 ± 41^a^^b^^e^	200	198 ± 41^a^^b^^e^	292	176 ± 35^a^^b^^c^^d^
SBP (mmHg)	525	127 ± 13^e^	775	125 ± 14^c^^e^	513	127 ± 16^b^^e^	204	125 ± 15^e^	311	121 ± 15^a^^b^^c^^d^

Data are mean and SD. BMI, body mass index. SBP, systolic blood pressure.

a, P<0.05 vs 1970s.

b, P<0.05 vs 1980s.

c, P<0.05 vs 1990s.

d, P<0.05 vs 2000s.

e, P<0.05 vs 2010s.

**Table 2:** Prevalence of CVD risk factors for women and men across decades.

	1970s		1980s		1990s		2000s		2010s	
	M	W	M	W	M	W	M	W	M	W
Obesity (%)*	13	11	22	25	38	34	46	43	35	44
HTN (%)^	31	13	28	16	34	26	42	26	30	25
HTN Medication (%)*	7	5	10	10	12	14	30	24	25	24
Diabetes (%)*	2	1	4	2	6	2	11	9	5	8
Diabetes Medication (%)*	1	0	1	1	2	1	8	6	5	7
Dyslipidemia (%)*	71	53	38	18	68	37	62	48	44	36
Dyslipidemia Medication (%)*	1	0	1	1	4	2	16	12	19	20
Smoking (%)*	22	11	18	12	10	8	8	8	7	3
Physical Inactivity (%)*	71	83	65	77	76	78	70	79	52	66

* P<0.05 for linear trend for men and women.

^P<0.05 for linear trend for women only. HTN, hypertension. M, Men. W, Women.

For men, age was higher (P<0.05) during 2000s than all other decades and was higher (P<0.05) in 1990s compared to 1970s, 1980s, and 2010s. BMI was lower (P<0.05) in 1970s and 1980s compared to the other three decades and was highest (P<0.05) during 2000s. Prevalence of obesity increased (P<0.05) across decades from 1970s to 2000s (Table 2). Blood glucose was lower (P<0.05) in the 1970s compared to all other decades. Prevalence of diabetes was highest (P<0.05) in the 2000s (Table 2). Total cholesterol declined (P<0.05) across decades from the 1970s to the 2010s and was lower (P<0.05) in the 2010s compared to all other decades. Resting systolic blood pressure was lower (P<0.05) during the 2010s relative to all other decades. There was no significant trend (P = 0.097) for prevalence of hypertension (Table 2).

Smoking prevalence decreased (P<0.05) across decades from the 1970s to 2010s and prevalence of physical inactivity was lowest (P<0.05) in the 2010s for both men and women (Table 2).

### Cardiorespiratory fitness

**Fig 1:**
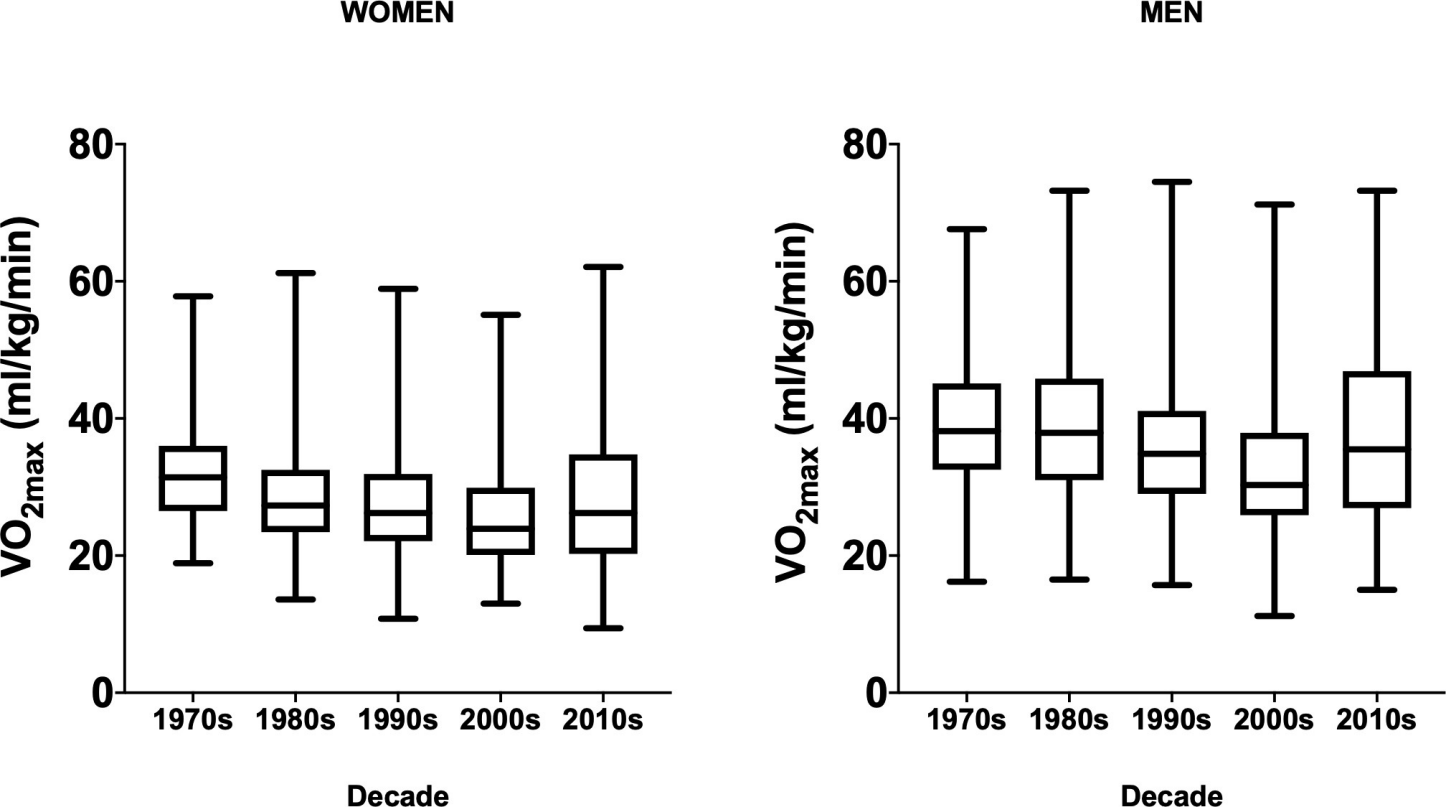
**Box and whisker plots of relative VO**_**2max**_ **for women (left) and men (right).** The box ranges from the 1^st^ quartile to the 3^rd^ quartile. The median is indicated by the line across the box. The whiskers start at the 1^st^ or 3^rd^ quartile and respectively end at the minimum or maximum values.

**Fig 2:**
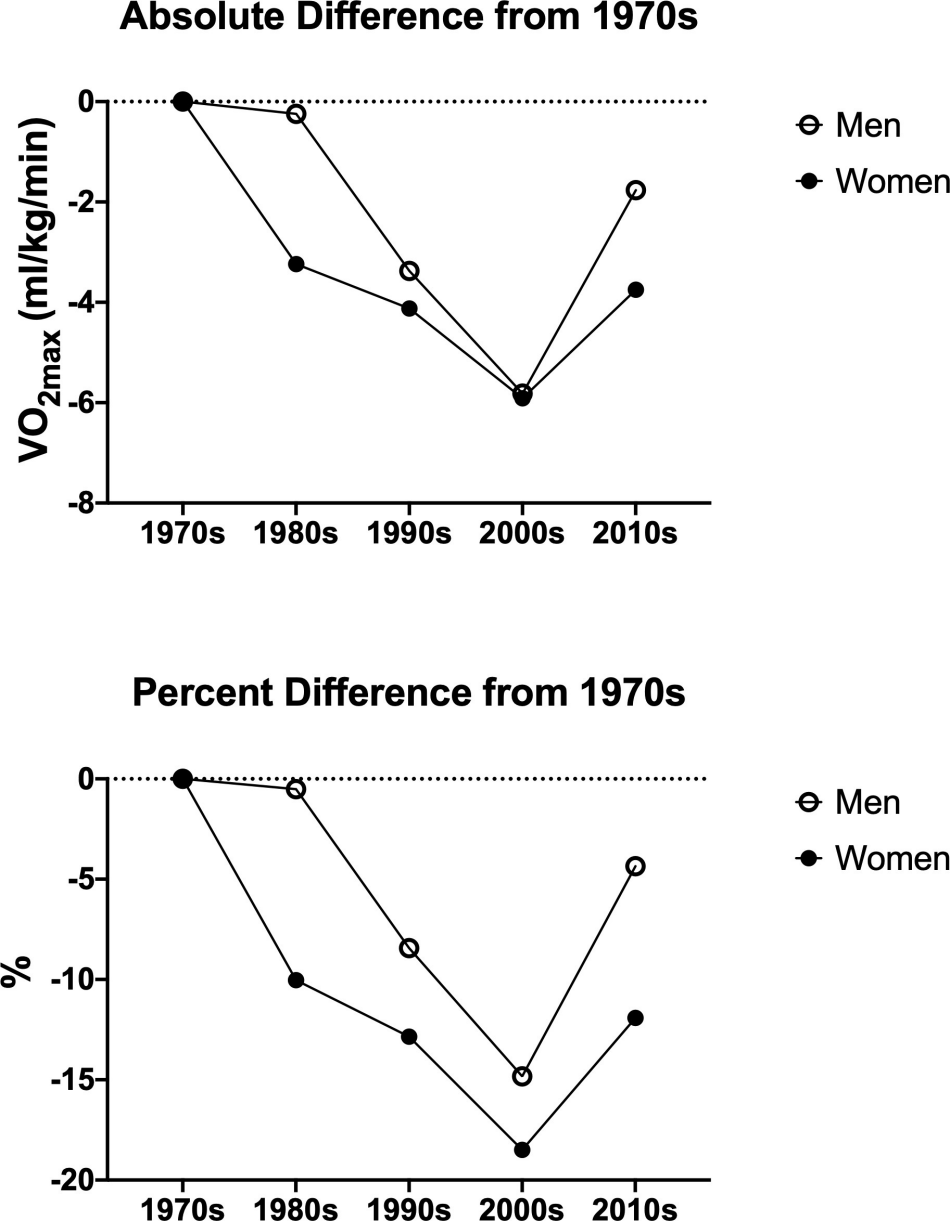
**Differences in CRF (i.e., relative VO**_**2max**_**) by decade in comparison to 1970s expressed as absolute difference (top) and percent difference (bottom) for women and men.** Values were determined from the means for each decade as presented in Table 3.

Maximal heart rate and three different expressions of cardiorespiratory fitness are presented in Table 3. For women, absolute VO_2max_ was higher (P<0.05) in the 2010s compared to the 1980s and 2000s. Relative VO_2max_ was higher (P<0.05) during the 1970s than all other decades and was lower (P<0.05) in the 2000s compared to the 1970s, 1980s, and 2010s (Figs 1 and 2). This finding remained significant (P<0.05) after adjusting for age differences across the decades. There was a significant (P<0.05) linear decline across the decades for relative VO_2max_. Age- and sex-adjusted FRIEND percentile was also higher (P<0.05) during the 1970s compared to all other decades and was lowest (P<0.05) during 2000s. Maximal heart rate, expressed as percent of age-predicted max, was not different between decades (P = 0.44).

**Table 3:** Maximal exercise test variables for women (N = 1824) and men (N = 2390) by decade.

	1970s	1980s	1990s	2000s	2010s
**Women**					
n	203	532	505	251	333
VO_2max_ (L/min)	2.03 ± 0.50	1.94 ± 0.46^e^	1.97 ± 0.46	1.93 ± 0.5^e^	2.06 ± 0.59^b^^d^
VO_2max_* (ml/kg/min)	31.9 ± 7.3^b^^c^^d^^e^	28.7 ± 7.8^a^^d^	27.8 ± 8.1^a^	26.0 ± 8.1^a^^b^^e^	28.1 ± 9.9^a^^d^
FRIEND* (%)	57 ± 24^b^^c^^d^^e^	50 ± 27^a^^d^	47 ± 26^a^	43 ± 27^a^^b^	47 ± 27^a^
HR_max_ (%)	100 ± 6	99 ± 8	100 ± 8	100 ± 7	99 ± 8
**Men**					
n	544	779	514	209	344
VO_2max_ (L/min)	3.19 ± 0.73	3.21 ± 0.75^d^	3.19 ± 0.73	3.05 ± 0.84^b^^e^	3.27± 0.86^d^
VO_2max_*(ml/kg/min)	39.1 ± 9.6^c^^d^	38.9 ± 10.5^c^^d^	35.8 ± 9.3^a^^b^^d^	33.3 ± 11.3^a^^b^^c^^e^	37.4 ± 12.5^d^
FRIEND* (%)	47 ± 27^c^^d^^e^	47 ± 28^c^^d^^e^	41 ± 26^a^^b^	40± 28^a^^b^	41 ± 26^a^^b^
HR_max_ (%)	101 ± 6^e^	101 ± 7^c^	102 ± 8^b^^e^	101 ± 9	99 ± 7^a^^c^

Data are mean and SD. FRIEND, Fitness Registry Importance of Exercise National Database. HRmax, percent of age-predicted max heart rate.

*P<0.05 for linear trend.

a, P<0.05 vs 1970s.

b, P<0.05 vs 1980s.

c, P<0.05 vs 1990s.

d, P<0.05 vs 2000s.

e, P<0.05 vs 2010s.

For men, absolute VO_2max_ was lower (P<0.05) in the 2000s compared to the 1980s and 2010s. Relative VO_2max_ was higher (P<0.05) during the 1970s and 1980s compared to 1990s and 2000s and was lower (P<0.05) in 2000s compared to all other groups (Figs 1 and 2), and remained significant (P<0.05) after adjusting for age. There was a significant (P<0.05) linear decline across the decades for relative VO_2max_. Age- and sex-adjusted FRIEND percentile was higher (P<0.05) during the 1970s and 1980s compared to the 1990s, 2000s, and 2010s. Maximal heart rate, expressed as percent of age-predicted max, was higher (P<0.05) in the 1990s compared to 1980s and 2010s.

For women, CRF across the decades was associated with the prevalence of physical inactivity (beta = -0.326, p<0.001), smoking (beta = -0.045, p = 0.019), obesity (beta = -0.331, p<0.001), dyslipidemia (beta = -0.090, p<0.001) and hypertension (beta = -0.224, p<0.001), but not diabetes (beta = -0.034, p = 0.082). For men, CRF across the decades was associated with the prevalence of physical inactivity (beta = -0.333, p<0.001), smoking (beta = -0.122, p<0.001), obesity (beta = -0.290, p<0.001), dyslipidemia (beta = -0.144, p<0.001), hypertension (beta = -0.174, p<0.001), and diabetes (beta = -0.096, p<0.001).

## Discussion

The primary purpose of this study was to assess trends in directly-measured CRF over the past 50 years (1970–2019) in apparently healthy adult men and women from the BALL ST cohort. This investigation revealed a general decline in CRF from 1970 through the 2000s and these differences could not be accounted for solely by differences in age across the decades. This pattern was generally associated with the prevalence of obesity and rates of physical inactivity over the same time period. These data suggest that the growing burden of CVD over the past five decades may be associated with decreases in CRF among adults without known or suspected CVD.

Given the established cardioprotective effects of CRF, global changes in CRF levels could have a profound effect on population health. The strong, independent, inverse relationship between CRF and mortality was initially reported by the Aerobics Center Longitudinal Study in 1989 [1] and has since been extensively established in diverse populations as a powerful predictor of CVD, CVD-specific and all-cause mortality [2–5]. We have previously shown that every 1-metabolic equivalent (MET) higher CRF value is associated with a 16% reduction in CVD mortality, even after adjustment for traditional CVD risk factors [20]. Thus, the observed CRF reduction of ~2-METs from 1970 through 2000s may have significant ramifications for future CVD burden. In support of this, CRF was associated with rates of hypertension in both men and women and diabetes in men, which is consistent with previous research [21, 22] [23–25], and suggests a higher risk of future CVD [26, 27].

When considering the mean CRF in our cohort during the 1970s and 2000s, the difference in CRF we observed was comparable to the decline recently reported by Kelly, et al. [10] over a similar time period in individuals referred for exercise testing and slightly higher than the decline in an international population assessment of over 2.5 million adults [7]. Not all studies have reported a decline in CRF as Knapik et al [28] observed no change in CRF in Army recruits from 1975–1998 and reports from the Cooper Clinic showed increases in CRF over time, particularly from 1970s to the 1980s [11, 12]. Differences in cohort demographics and geographical location are likely explanations for the contrasting results and highlights the importance of examining trends in multiple diverse cohorts. The increase in CRF over time in the Cooper Clinic studies was associated with higher levels of physical activity [11, 12]. Indeed, physical activity, particularly in the form of moderate and vigorous intensity exercise, is closely associated with CRF and is the primary intervention for improving CRF (reviewed in [5]) as was the case in the current cohort. Thus, following global recommendations to perform physical activity regularly, especially in the form of exercise with sufficient intensity, is key to improving CRF which will in turn confer valuable health benefits [29, 30].

The BALL ST cohort is comprised of apparently healthy adults that self-referred to a university-based exercise program or for fitness testing and individuals that volunteered for health-related research. As evidenced by the age- and sex-adjusted FRIEND score, women had above average CRF in the 1970s and progressively declined to below average (50^th^ percentile) through the 2000s while men were slightly below average in the 1970s and remained below average throughout the 50 years. An optimistic observation from these trends is that CRF did not decline over the most recent decade, which was associated with lower rates of physical inactivity in both women and men and lower prevalence of obesity in men. This reduction in prevalence of physical inactivity over the past decade is consistent with data from the National Health Interview Survey as presented in the 2019 Heart Disease and Stroke Statistics Update [31]. Further, this coincides with an increase in the prevalence of adults meeting the aerobic exercise recommendations [29, 31]. Due to the strong association among physical activity, CRF, and CVD, these trends suggest a potential future reduction in CVD. However, this optimism is tempered by trends of declining CRF in children [6, 8] combined with lower percentage of High School students meeting the physical activity guidelines [29]. Thus, it is imperative for the prevention of future CVD, that efforts are made to promote and improve physical activity levels and CRF in children, adolescents, and adults [32, 33].

There are several strengths and limitations that should be considered when interpreting these results. This was a cross-sectional study design of individuals who self-referred for an exercise program, fitness testing, or health-related research and thus demonstrated an interest in exercise and/or fitness. However, as noted by the FRIEND score, our cohort had roughly average or below average CRF levels compared to national averages. We assessed CRF using peak exertion cardiopulmonary exercise testing which is the gold-standard method and provides an accurate objective measure. This is notable as estimated CRF from exercise test parameters or using non-exercise prediction equations has an error >1-MET and only correctly categorizes CRF level in ~50% of individuals [34]. The BALL ST cohort is 95% non-Hispanic whites from a relatively small area of the Midwest region of the United States and thus generalization of results may not apply to the entire population. Importantly, the cohort does include both men and women across a wide range of ages (18–90 years) and CRF levels (Fig 1) and CVD risk factor profiles. The association between CRF and risk of myocardial infarction and mortality is independent of race [35, 36], however, race is known to influence CRF [37] and CRF is apparently higher in White compared to Black men and women [35, 38]. Thus, it is necessary to examine trends in CRF overtime in diverse populations varying in race and ethnicity.

In conclusion, CRF was highest during the 1970s and lowest during the 2000s in apparently healthy adult men and women that self-referred for fitness assessment. The differences in CRF over the past 50 years are associated with changes in obesity and physical inactivity and a worsening CVD risk profile. Given the strong association between CRF and CVD, it is important to monitor CRF in all patients as part of routine clinical practice [5]. Further, preventative efforts to reduce the global burden of CVD should include physical activity with an emphasis on improving CRF.
